# Effectiveness of Magnolol, a Lignan from Magnolia Bark, in Diabetes, Its Complications and Comorbidities—A Review

**DOI:** 10.3390/ijms221810050

**Published:** 2021-09-17

**Authors:** Katarzyna Szałabska-Rąpała, Weronika Borymska, Ilona Kaczmarczyk-Sedlak

**Affiliations:** 1 Doctoral School of the Medical University of Silesia in Katowice, Discipline of Pharmaceutical Sciences, Department of Pharmacognosy and Phytochemistry, Faculty of Pharmaceutical Sciences in Sosnowiec, Medical University of Silesia, Katowice, Jagiellońska 4, 41-200 Sosnowiec, Poland; 2 Department of Pharmacognosy and Phytochemistry, Faculty of Pharmaceutical Sciences in Sosnowiec, Medical University of Silesia, Katowice, Jagiellońska 4, 41-200 Sosnowiec, Poland; wwojnar@sum.edu.pl (W.B.); isedlak@sum.edu.pl (I.K.-S.)

**Keywords:** magnolol, lignan, diabetes, diabetic complications, plant-derived polyphenols, antidiabetic potential

## Abstract

Diabetes mellitus is a chronic metabolic disease characterized by disturbances in carbohydrate, protein, and lipid metabolism, often accompanied by oxidative stress. Diabetes treatment is a complicated process in which, in addition to the standard pharmacological action, it is necessary to append a comprehensive approach. Introducing the aspect of non-pharmacological treatment of diabetes allows one to alleviate its many adverse complications. Therefore, it seems important to look for substances that, when included in the daily diet, can improve diabetic parameters. Magnolol, a polyphenolic compound found in magnolia bark, is known for its health-promoting activities and multidirectional beneficial effects on the body. Accordingly, the goal of this review is to systematize the available scientific literature on its beneficial effects on type 2 diabetes and its complications. Taking the above into consideration, the article collects data on the favorable effects of magnolol on parameters related to glycemia, lipid metabolism, or oxidative stress in the course of diabetes. After careful analysis of many scientific articles, it can be concluded that this lignan is a promising agent supporting the conventional therapies with antidiabetic drugs in order to manage diabetes and diabetes-related diseases.

## 1. Diabetes Mellitus—A Global Public Health Problem

Diabetes mellitus, or in short diabetes, is a chronic metabolic disease characterized by a disturbed carbohydrate, lipid, and protein metabolism. In this disease an increased production of reactive oxygen species (ROS) is observed. This leads to oxidative stress, which is defined as the imbalance between the production and neutralization processes of ROS in the body. Chronic persistence of oxidative stress consequently leads to numerous diabetic complications in an exhausted organism, as well as retinopathy, nephropathy, cardiovascular and peripheral vessels disorders (including micro- and macro-vascular changes) or neuropathies [1,2,3,4].

Most frequently, diabetes is divided into two major types: type 1 and type 2. In type 1 diabetes, the β-cells of the pancreas are irreversibly damaged, and thus insulin secretion is impaired. To cope with this type of diabetes, patients must take exogenously administered insulin. Worldwide, type 2 diabetes is the most widespread (about 85% of the diabetic population suffers from the disease) and it is associated with insulin resistance and a decrease in the sensitivity of individual tissues of the body to the action of this hormone. The most preferred treatment for type 2 diabetes is therapy with oral antidiabetic agents. Apart from these two major types of diabetes, other types, such as gestational diabetes and diabetes of unknown etiology, are also mentioned [5,6]. In 2009, approximately 285 million people suffered from diabetes. This number increased to 366 million in 2011, and in 2019 it was over 463 million. If the upward trend in the incidence of this disease continues, it is expected that in 2030 up to 578 million people will have diabetes, and by 2045 reaching 700 million. Furthermore, another problem is the detection of this illness; currently, about half of people with diabetes is unaware that they suffer from this disease. Its late detection is associated with the manifestation of numerous serious complications, a more difficult treatment process, higher mortality, and higher financial costs of treating such patients [7].

The multidirectional approach to diabetes seems to be significant in the process of managing this disease. The benefits of comprehensive approach were proven in a large randomized clinical trial in type 2 diabetic kidney disease [8]. The study highlighted the collective negative impact of comorbidities of a patient and the fact that they can lead to the development of other serious medical conditions, which together may contribute to overall higher mortality. Interestingly, supplementing standard pharmacotherapy with non-pharmacological activities such as: lifestyle changes, changing eating habits, or increasing physical activity, improved the results of the patients suffering from diabetic kidney disease and resulted in a better prognosis for survival. Taking the above into consideration, it seems crucial to look for additional therapeutic elements supporting standard treatment of type 2 diabetes [8].

## 2. Magnolol—A Brief Description of a Chemical Compound, Its Occurrence in Nature and the Most Important Activities

**Figure 1:**
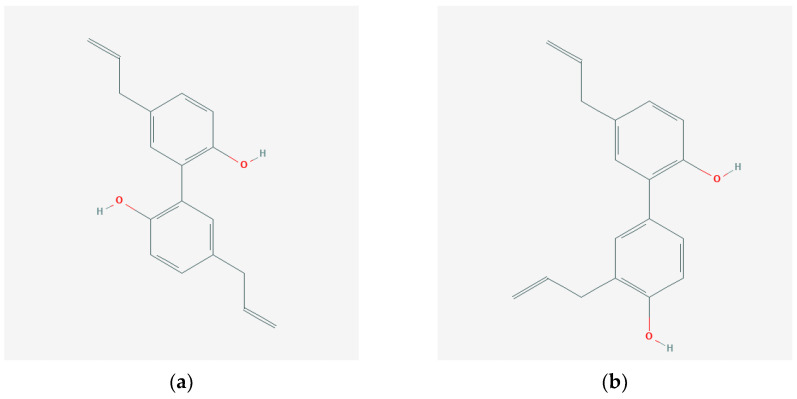
Structural formulas of magnolol (**a**) [19] and its optical isomer—honokiol (**b**) [20].

Magnolol Figure 1a is a chemical compound classified in the specific group of polyphenols called lignans. Magnolol occurs in the bark of various types of magnolia, including: *Magnolia officinals*, *Magnolia obovata,* or *Magnolia dealbata*, and is often accompanied by another lignan, which is its optical isomer, honokiol Figure 1b [9,10,11,12]. In traditional Chinese medicine, magnolia bark is considered to have antipyretic, analgesic, sedative, constipating, anti-asthmatic, cardioprotective, vasoprotective, or antibacterial properties. Numerous scientific studies report that the biological activity of magnolol includes antioxidant, anti-inflammatory, anti-cancer, analgesic, neuroprotective, anticoagulant, and smooth muscle relaxing effects. There are also reports of its impact on glycemic control and elimination of the effects of diabetic complications [10,11,12,13,14,15,16,17]. Despite the fact that magnolol and honokiol are optical isomers, scientific works on their biological activity indicate their slightly diverse activities in vitro and in vivo.

As the type 2 diabetes is the predominant type of diabetes worldwide, the aim of this article is to review the literature and present the current state of knowledge regarding the effect of magnolol on the course of type 2 diabetes and its complications in laboratory in vivo models. Additionally, the influence of magnolol on the diabetes-related parameters examined in the in vitro studies is also presented in this review. Most of the articles cited in this review encompass type 2 diabetes and its complications. The few cases that are related to type 1 are clearly highlighted in the text.

A detailed review regarding numerous properties of magnolol as well as its bioavailability, toxicity and pharmacology has been recently published by Lin Yiping [18]. Nevertheless, our work primarily focuses on the influence of magnolol on glucose homeostasis and glycemic control, as well as its effect on disrupted lipid metabolism in the course of diabetes and the neutralization of the effects of diabetic complications in various organs. To present the therapeutic effect of magnolol on type 2 diabetes, its complications and comorbidities, the scientific literature databases Google Scholar, PubMed, Embase, and Cochrane were reviewed. For this purpose, the following keywords and their combinations were used: magnolol, *Magnolia*, diabetes, hyperglycemia, oxidative stress, nephropathy, retinopathy, neuropathy, cardiovascular disease, obesity, reproductive system, ocular complication, diabetic foot, testes, ovaries, inflammation, lipid profile, glucose, insulin, or intestinal microflora.

## 3. Magnolol in Diabetes and Its Complications

### 3.1. Magnolol in Glycemic Control

In diabetes, cytochrome P450 2E1 (CYP2E1) overexpression is observed at protein and mRNA levels. Increased CYP2E1 expression in diabetes is related to increased production of ROS and, consequently, the formation of oxidative damage. It is believed that ROS produced by this cytochrome may also contribute to the development of insulin resistance in the body, both in the course of diabetes and other metabolic diseases. This is due to impairment of insulin signaling pathways, contributing insufficient uptake glucose from adipocytes [21,22]. In hepatocytes, disruption of insulin signaling pathways for insulin receptor substrates (IRS-1 and IRS-2), phosphatidylinositol 3-kinase (PI3K) and protein kinase B (PKB, which also known as Akt) leads to the development of CYP2E1-dependent cytotoxicity [21,23,24]. Considering the role of cytochrome CYP2E1 in the pathological formation of ROS, the study conducted on high fat diet (HFD)-induced diabetic rats proved that oral administration of magnolol (at doses of 25, 50 and 100 mg/kg) decreased the activity of hepatic CYP2E1, which ultimately contributed to the reduction of insulin resistance in the group of tested rats. In the same study the authors performed oral glucose tolerance test (OGTT) in the rats and concluded, that after magnolol treatment, regardless the dose, the area under the curve (AUC) was lower than AUC obtained in untreated animals. The serum glucose level was also reduced [21]. Studies conducted on mice indicate that knockout of CYP2E1 may increase the body’s sensitivity to insulin and protect it against glucose intolerance and obesity caused by a HFD [25]. It is worth mentioning that in the study conducted on HFD mice that received magnolol or mixtures of magnolol and honokiol, the OGTT in both cases also showed lower AUC, in comparison to the untreated mice [26]. Furthermore, magnolol administered to rats with type 2 diabetes reduced fasting blood glucose and plasma insulin levels, without affecting their body weight [27].

One of the type of drugs used in the treatment of diabetes mellitus is a group of substances called α-glucosidase inhibitors (AGIs). This group includes acarbose, voglibose, and miglitol. Their mechanism of action is based on the inhibition of the α-glucosidase enzyme, which is responsible for the selective hydrolysis of terminally linked α-glucose residues from complex carbohydrates in the brush border of the small intestine. As a result of the competitive attachment of drugs from the AGIs group to the α-glucosidase carbohydrate binding site, the absorption of the glucose molecule is delayed. Therefore, glucose undergoes gradual hydrolysis in the further segments of the small intestine and, thus, a reduced blood glucose level is obtained [28,29,30].

There are reports indicating that substances of phenolic origin may be a potential new class of AGIs. One of such reports focused on investigating the α-glucosidase inhibitory potential of magnolol-derived dimeric neolignans. Mentioned study proved that magnolol derivatives show a potent ability to inhibit α-glucosidase. Although inhibitory activity towards α-glucosidase of magnolol was not as strong as some of these neolignans, its ability to inhibit α-glucosidase was higher than its optical isomer, honokiol, and standard substances (quercetin and acarbose) [31].

Methylglyoxal (MGO) is one of various parameters that allows one to detect a diabetic state. Its increased concentration in blood plasma is often observed among patients with uncontrolled diabetes. MGO contributes to the formation of an increased amount of advanced glycation end products (AGEs) by interacting with the amino acid side chains in selected proteins. Additionally, enhanced formation of AGEs may cause excessive self-oxidation of glucose, resulting in the formation of glyoxal and then, after fragmentation, MGO [32,33]. In a healthy organism, there are protective mechanisms which compensate the harmful effects of MGO on the cells. Oxidative stress is one of the factors weakening the defense ability of the body cells against exogenous and endogenous pathogens. It is believed that it is responsible for the reduction of the mass of pancreatic islet β cells and progressive process of their destruction in the course of diabetes. Pancreatic β cells are extremely sensitive to ROS [34,35,36]. It is proven that the increased concentration of MGO results in the excessive production of ROS in the body. The consequence of the excessive production of ROS during oxidative phosphorylation is the up-regulation of mitochondrial uncoupling protein 2, leading to electron leakage and reduction of ATP synthesis. These processes potentially cause disruptions in insulin secretion in the cells of the pancreas [35,37,38]. Further, ROS contribute to the increased self-oxidation of unsaturated fatty acids and proteins of the cytoplasmic membrane [34,39], which leads to increased production of malondialdehyde (MDA), a compound that is another marker of the occurrence of oxidative stress in the body [40]. Chronically persistent hyperglycemia promotes the occurrence of oxidative stress, which entails a number of pathological changes in the body, including impaired insulin secretion or excessive apoptosis of β cells of the pancreatic islets and in the long run leads to overt manifestation of diabetes [35,36,37]. An in vitro study conducted on rat pancreatic β-cells after 48-h exposure to MGO showed a significant loss of viability and impaired insulin secretion. On the other hand, administration of magnolol in concentrations of 0.01–1.0 µM resulted in an increase in insulin secretion by the cells, the decrease in cell apoptosis and the increase in their viability. After pretreatment with magnolol, there was also the increase in the expression of genes such as insulin 2 and pancreatic and duodenal homeobox 1, which are responsible for the survival and proper functioning of pancreatic islet β cells. Moreover, magnolol positively influenced the activation of the adenosine monophosphate-activated protein kinase (AMPK)/sirtuin 1/peroxisome proliferator-activated receptor γ coactivator-1-α pathway, which plays a key role in the regulation of mitochondrial biogenesis. The experiment confirmed that magnolol protects the cells against excessive protein glycation induced by MGO via increasing the activity of glyoxalase (an enzyme that decomposes MGO) and lowering the level of MGO modified protein adducts [35].

The protein tyrosine phosphatase-1B (PTP1B) belongs to the group of non-transmembrane phosphotyrosine phosphatases, expressed in insulin-targeted tissues. It plays the role of a negative regulator of the insulin signaling pathway and therefore the potential use of PTP1B inhibitors may result in the improvement of some diabetic parameters [41,42]. In vitro studies showed that magnolol is an inhibitor of PTP1B, which inhibits the expression of this phosphatase in a dose dependent manner. Based on the Lineweaver–Burk plot and molecular docking, it was possible to determine that magnolol is a non-competitive PTP1B inhibitor. More importantly, it exhibited enzymatic selectivity for PTP and promoted cellular activity in the insulin signaling pathway [43]. A summary of the effect of magnolol on the parameters related to glucose and insulin homeostasis in vitro can be found in Table 1 and related to glucose, insulin and lipid homeostasis, as well as to oxidative stress, inflammation, and molecules glycation in vivo in Table 2.

A brief review regarding the prevention of diabetic complications with extracts of *Magnolia* species was also presented by Zhao Xuezhong [17].

### 3.2. Magnolol and Diabetic Nephropathy

Diabetes mellitus is a major contributor to the development of chronic kidney disease, and diabetic nephropathy is one of the leading causes of death among people with diabetes [17]. Diabetic nephropathy is accompanied by the loss of kidney function and increased excretion of protein in the urine. The most frequently mentioned factors responsible for the development of this disease are increased blood glucose level as well as excessive production of AGEs [27,47,48,49,50], transforming growth factor β (TGF-β) [27,48,49,50] or sorbitol accumulation [27,50]. Moreover, hyperglycemia and AGEs stimulate TGF-β, which influences the production of individual matrix components [27,47,48,49,50] such as: fibronectin, collagen, laminin or proteoglycans in the kidneys. In patients suffering from diabetes, the activity of aldose reductase, an enzyme converting glucose to sorbitol, is also increased as a result of the accumulation of glucose in the body. Activation of the sorbitol pathway is closely related to the increased production of TGF-β1 [27,51]. As a result of all pathological changes at the cellular level, that are observed in diabetic nephropathy, several symptoms are likely to develop. The latter include glomerular hyperfiltration, glomerular hypertension and renal hypertrophy, as well as, thickening of the glomerular basement membrane, proliferative changes and atrophy of the renal tubules, and finally interstitial fibrosis and glomerular sclerosis [47,52]. Administration of magnolol at a dose of 100 mg/kg to Goko-Kakizaki rats improved the parameters of diabetic nephropathy and glucose homeostasis, such as blood glucose level, plasma insulin level, creatinine clearance, urinary protein, kidney sorbitol, and AGEs level. In addition, magnolol impaired the overregulation of mRNA for kidney TGF-β1 and furthermore diminished the expression of type IV collagen of extracellular matrix proteins. All of these factors lead to the amelioration of renal dysfunction resulting from diabetic nephropathy [27]. KIOM-79 is an ethanolic mixture of plant extracts obtained from the pueraria root, magnolia bark, liquorice root, and spurge root. HPLC analysis showed that the main components of KIOM-79 are puerarin, glycyrrhizin, magnolol, and its optical isomer honokiol [53]. The use of KIOM-79 in stopping the progression of diabetic nephropathy in Zucker fatty rats was investigated. In the course of that study, it turned out that KIOM-79 had a positive effect on the state of diabetic nephropathy by reducing blood glucose level, albuminuria, glomerular sclerosis, degeneration of the renal tubules or podocyte apoptosis, and reducing the accumulation of AGEs or protein oxidation in the glomeruli, as well as, TGF-β1 in renal cortex [54]. The positive effect of KIOM-79 on diabetic nephropathy was also demonstrated in type 2 diabetic Goko-Kakizaki rats. Administration of KIOM-79 to animals at a dose of 500 mg/kg for 13 weeks resulted in decreased blood glucose and plasma insulin levels, formation of AGEs in the glomeruli and overexpression of mRNA for renal type IV collagen, TGF-β1 and vascular endothelial growth factor (VEGF) [55]. Inhibitory effect of KIOM-79 on AGEs formation and expressions of type IV collagen and TGF-β1, but in type 1 diabetes, was also investigated. In this particular study KIOM-79 administration to streptozotocin (STZ)-induced diabetic rats resulted in the decrease in mesangial matrix and glomerular volume, as well as reduction in overexpression of renal mRNA for type IV collagen and TGF-β1. The same research also confirmed the positive effect of KIOM-79 on the inhibition of AGEs formation in vitro [56]. AGEs formation inhibition was also indicated in vitro by unprocessed and processed magnolia bark extract containing magnolol [57]. The beneficial effects of magnolol on the kidneys were mentioned likewise in other, non-diabetic research models that analyzed other markers of kidney damage related to apoptosis and oxidative stress [58,59]. Antidiabetic activity and the effect of *Magnolia* extracts on the basic parameters of oxidative stress in in vitro model lines can be found in Table 3a and antidiabetic activity and the effect of *Magnolia* extracts on the basic parameters of oxidative stress and lipids in in vivo animal models can be found in Table 3b.

### 3.3. Magnolol and Diabetic Neuropathy

To the best of our knowledge, there are currently no studies describing the neuroprotective properties of magnolol in relation to degenerative changes occurring in the course of diabetes mellitus such as neuropathy, disorders of autonomic functions or the diabetic foot. Despite the lack of studies on the neuroprotective effect of magnolol in diabetes, there are many reports about its positive effect on the functioning of the nervous system [15,16,18]. Magnolol in in vivo studies on animal models showed a protective effect in Parkinson’s disease [65] and Alzheimer’s disease [66], supported the processes of concentration and learning and improved memory [66,67], or revealed antidepressant activity [68,69]. It should also be emphasized that magnolol has a proven analgesic effect [70,71]. Due to numerous reports on the protective effect of magnolol on the nervous system, it can be assumed that this lignan may also have neuroprotective effects in the course of diabetes, but careful research in this area is required.

### 3.4. Magnolol and Retinopathy and Eye Diseases Related to Diabetes

Diabetic cataract is one of the complications that affects people with chronic diabetes. As a result, the cells of the lens epithelium are damaged, and then the lens becomes cloudy and the functionality of the eye is impaired. The factors that are considered to be the causative agents of cataract include: activation of the polyol pathway, excessive production of AGEs or the occurrence of oxidative stress [53,72,73]. AGEs are also responsible for excessive apoptosis in the pericytes of the retina, corneal endothelial cells, or neuronal cells, and the mechanism of this phenomenon is the induction of oxidative stress and increased expression of pro-apoptotic cytokines [53,73]. Additionally, activation of nuclear factor kappa-light-chain-enhancer of activated B cells (NF-κB) in the lens epithelial cells was observed in vitro [74]. Another pathological change that is observed in both the course of cataract and other eye diseases (including retinitis, uveitis, glaucoma) is overexpression of induced nitric oxide synthase (iNOS) [53,74,75,76]. Under physiological conditions, nitric oxide (NO) is responsible for neurotransmission or vasodilation; it is a specific signaling molecule. However, its excessive production associated with the activation of iNOS by cytokines, NF-κB [77] or AGEs, leads to pathological changes within the eye [53,74]. The in vitro hyperglycemia-induced cataract model showed that the use of NOS inhibitors improved the condition of the eye cells [78]. Thus, substances showing the ability to inhibit iNOS activity, production of AGEs and overexpression of NF-κB are potentially promising agents protecting the eye against diabetic complications [53].

It is worth noting that AGEs may also be responsible for other mechanisms related to eye pathologies in the course of diabetes [79]. AGEs can lead to increased expression of extracellular matrix proteins (type IV collagen, fibronectin, laminin) that mediate the development of diabetic complications mainly in association with the growth factor TGF-β [44,51,80]. In relation to diabetic retinopathy, these phenomena lead to a thickening of the vascular basal membrane and disturbance of normal vision [81]. TGF-β itself is responsible for the processes of differentiation, proliferation, migration or apoptosis of extracellular matrix proteins, and in the cells of the eye tissue for the pathophysiological processes of its development and repair. In diabetic retinopathy, TGF-β plays a key role in neovascularization by stimulating VEGF secreted by retinal pigment epithelial cells. These cells are located between the retina and the choroid, forming the outer blood-retinal barrier, and in pathological processes occurring within the eyeball lead to progressive loss of vision [44,82,83,84].

As it was mentioned before, KIOM-79 is an ethanolic blend, which includes, among others, magnolia bark. The combination of compounds contained in this blend has the ability to delay the development of diabetic cataract and significantly inhibited lens epithelial cell apoptosis by reducing AGEs production, NF-κB activation, and iNOS expression. KIOM-79 induced also a minor decrease in blood glucose level. Magnolol is considered to be a factor preventing the oxidation of low-density lipoprotein (LDL), responsible for inducing apoptosis of vascular endothelial cells, and honokiol, a factor inhibiting the activation and regulating the expression of genes responsible for the activation of NF-κB and reducing apoptosis of vascular endothelial cells. These actions can explain some of the positive aspects of KIOM-79 therapy and the improvement of the functionality of the visual organ in Zucker fatty rats by inhibiting lens opacity [53].

The above mentioned KIOM-79 was also tested in vivo in rat model of diabetic keratopathy. This disease is another diabetic complication that affects the quality of vision and is able to damage the cornea of the eye [85,86]. AGEs bear the responsibility for this condition [87]; their toxic effect is due to their ability to cause structural and functional alterations in the plasma and the extracellular matrix proteins [62]. Moreover, interaction between AGEs and their receptors cause enlarged formation of ROS, which afterwards activate NF-κB and release pro-inflammatory cytokines [88]. Studies on the Zucker fatty rat model, in which animals received KIOM-79, showed that this mixture had a positive effect on the cornea of animals and prevented destructive changes resulting from the action of oxidative stress. A decrease in AGEs level, 8-hydroxy-2′-deoxyguanosine (DNA damage marker) formation, NF-κB activity and Bax protein (marker of apoptosis) expression and minor decrease in blood glucose level were observed [62].

In vitro studies of human retinal pigment epithelial cells proved that application of magnolol up to 20 µg/mL for a period of 24 h caused no alterations in the viability of these cells. In the same study, the inhibitory effect on TGF-β1 expression was evaluated. The one-hour pretreatment with magnolol (in concentrations of 0.5–5 µg/mL) before incubation with high glucose, revealed that the increase in TGF-β1 protein secretion and high glucose level were reduced. Pretreatment with magnolol also resulted in the decreased mRNA expression for fibronectin and MDA level, as well as in inhibition of extracellular signal-regulated kinase/mitogen-activated protein kinase (MAPK)/Akt signaling and resulted in a decrease in TGF-β levels [44]. The positive effect of KIOM-79 on the prevention of apoptosis and cell death, as well as the accumulation of AGEs was demonstrated in retinas of diabetic C57BL/KSJ-db/db mice [64]. A summary of the effect of magnolol on the parameters related to oxidative stress, inflammation and molecules glycation in vitro can be found in Table 1.

Ischemic retinopathy is defined as a number of disorders that lead to loss of vision, including retinopathy in premature children, vascular obstruction, age-related macular degeneration or diabetic retinopathy. A characteristic lesion of ischemic retinopathy is retinal neovascularization and retinal glial cell dysfunction caused by overexpression of the VEGF [89]. An in vivo oxygen-induced retinopathy model (model of ischemic retinopathy) in C57BL/6J mice that were treated intraperitoneally with magnolol at the dose of 25 mg/kg for five days showed that magnolol partially influenced retinal neovascularization by inhibiting glial cell degradation and preventing microglial inflammatory responses induced by the signaling pathway of hypoxia-inducible factor 1a/VEGF [90].

### 3.5. Magnolol and Cardiovascular Complications in Diabetes

Numerous scientific studies indicate the therapeutic potential of magnolol on the cardiovascular system, however, none of these studies which has been published so far, has been performed on a type 2 diabetic model [15,16,17]. Nevertheless, we managed to find the study that was performed in type 1 diabetic model in which magnolol was administered to mice at the doses of 10 mg/kg or 20 mg/kg, respectively, for 12 weeks. It turned out that magnolol improved the overall condition of the heart tissue of type 1 diabetic mice. There was the decrease in cardiac failure parameters such as: creatine kinase, MB isoenzyme of creatine kinase and lactate dehydrogenase, as well as the extenuation of cardiac mRNA expression for tumor necrosis factor α (TNF-α) and interleukin 6 (IL-6). As a conclusion, the authors indicated that magnolol could attenuate the inflammation-mediated diabetic myocardial injuries via inhibiting activation of MAPK/NF-κB signaling pathway [91]. What is more, this lignan was found to have cardioprotective potential by inhibiting myocardial fibrosis, including excessive proliferation of cardiac fibroblasts and accumulation of extracellular matrix [92]. This disease accompanies both arterial hypertension, myocardial infarction, but also diabetic cardiomyopathy and heart failure [93]. In in vitro conditions, the decrease in cell proliferation and collagen synthesis was observed in the cell culture of cardiac fibroblasts, in which magnolol was applied in concentrations of 5–20 µM. Additionally, magnolol was found to be an agonist of the ALDH2 receptor—a key mitochondrial enzyme (aldehyde dehydrogenase) involved in the detoxification of reactive aldehydes and showed a protective effect against myocardial fibrosis [92]. In rats with essential hypertension, oral administration of magnolol at a dose of 100 mg/kg for three weeks, led to a hypotensive effect at the early stage of hypertension. This effect was obtained due to, among others, the improvement of vascular insulin resistance via upregulation of the peroxisome proliferator-activated receptor gamma (PPARγ), downregulation of thromboxane-3 and increasing the activity of PKB and endothelial NOS in blood vessels [94]. In hyperglycemic rabbits the extract obtained from *Magnolia officinalis* reduced oxidative stress and regulated plasma lipids, as well as decreased arterial lesions. This extract also down-regulated apoptosis-related gene expression in the aortic arches [95]. There is also a report about a beneficial effect of magnolia bark extract (and so a mixture of magnolol and honokiol) on various parameters of the circulatory system including reduction of lipid accumulation, limitation of inflammation and the effects of oxidative stress, and diminution of apoptosis in the hearts of mice on a HFD [96]. It was also proven that pure magnolol, both in vitro and in vivo, can have a beneficial effect on blood vessels and the heart muscle, as well as it can have a hypotensive effect and prevents ischemic changes by modulating various signaling pathways [96,97,98,99,100,101,102]. The above scientific reports may indicate a potential protective effect on the cardiovascular system, also in the course of type 2 diabetes, however, in order to prove this assumption, tests should be performed using an appropriate diabetic model.

### 3.6. Magnolol and Lipid Metabolism Disorders in Diabetes

Adipose tissue plays an important role in energy homeostasis, therefore, adipocytes are the main target of drugs used in obesity and metabolic syndrome [103,104]. In pathological obesity, adipocytes are enlarged and there is an increased secretion of TNF-α and plasminogen activator inhibitor-1 (PAI-1) [13,105,106], as well as pro-inflammatory chemokines and cytokines: monocyte chemoattractant protein 1, interleukins IL-6 and IL-10. The production of various adipokines including adiponectin, resistin and leptin is also changed [11,103,104,105,107]. It is worth noting that properly developed, small adipocytes have a significant impact on the regulation of energy metabolism in the body, especially by increasing its sensitivity to insulin [13,108,109]. Obesity is associated with an increased risk of metabolic diseases such as type 2 diabetes, atherosclerosis, dyslipidemia and fatty liver disease. Among obese people, insulin resistance is often observed, which is considered to be the main cause of the metabolic syndrome. In addition, obesity-induced inflammation in the body, often leading to adipose tissue dysfunction, is considered to be a factor that connects it with insulin resistance [11,103,106,110]. The most important nuclear receptors that regulate transcription through interaction with the appropriate ligand are PPARs. PPARγ receptors are abundant in the adipose tissue and have a significant impact on the differentiation of adipocytes [110,111,112]. PPARγ ligands are responsible for the increased transport of glucose through insulin-sensitive tissues by regulating the expression of genes involved in glucose metabolism. GLUT1 and GLUT4 are glucose transporters, with GLUT4 being an insulin-dependent transporter. GLUT 1 is expressed in both preadipocytes and mature adipocytes, and its level is independent of adipocyte differentiation. GLUT 4 expression occurs only in mature adipocytes and is regulated by PPARγ [13,111,113,114].

One of the strategies of diabetes and obesity treatment is a therapy with a suitable agonist of PPAR-γ. These include, among others, glitazones. It is proven that drugs from this group reduce the level of LDL, triglycerides (TG) and glucose in the blood plasma and improve the sensitivity to insulin in the organism by increasing insulin-dependent glucose uptake and inhibiting its production in the liver cells. The effect of glitazones on the improvement of diabetic parameters was studied in both animal model and in humans [26,115,116]. The PPARγ receptor is a nuclear receptor that can be activated by a specific ligand, forming a heterodimer with the retinoid X receptor (RXR), PPARγ-RXR. Such a combination ensures the maintenance of adequate homeostasis of the body in relation to the levels of glucose, lipids or the regulation of gene expression related to their metabolism. Ongoing research on substances that are direct ligands for the RXR receptor is now emerging. RXR ligands are able to activate the PPARγ-RXR heterodimer and, consequently, achieve beneficial antidiabetic effects. Docking the ligand to the RXR receptor increases the chance of attaching co-activators to the PPARγ-RXR heterodimer, and some of the RXR ligands may increase the expression of genes targeted by PPARγ ligands. Accordingly, RXR ligands lower plasma glucose levels [26,111,112]. In the study conducted on a mouse model with obesity induced by a HFD, the animals were orally administered honokiol (100 mg/kg), magnolol (100 mg/kg) or a mixture of both these lignans (50 + 50 mg/kg). The obtained results revealed that the mixture of these two compounds showed the greatest effectiveness. It may be related to their synergistic action, enhancement of pharmacological action, as well as improvement of lipid (reduction of total cholesterol (TC) in the blood plasma) and glucose (reduction of its level in the blood plasma) metabolism. There are several effects of a mixture of honokiol and magnolol which are particularly important, that is: a reduction of the body weight gain, the decrease in the white fat weight and the increase in mRNA expression for PPARγ and targeted genes, such as GLUT4 and adiponectin in white adipose tissue. It should be highlighted that honokiol is a ligand of the RXR receptor, so it principally affects the regulation of cholesterol metabolism by upregulation of adenosine triphosphate (ATP)-binding cassette transporter expression and elevation of insulin-dependent glucose uptake in adipocytes. Magnolol, conversely, acts as a ligand for the PPARγ receptor and mainly influences the differentiation of adipocytes and causes an increase in insulin-dependent glucose uptake in them. While studying the effects of a mixture of magnolol and honokiol on the insulin signaling pathway in white adipose tissue, it turned out that this mixture increased glucose uptake [26]. The underlying mechanism of this action can be activation of insulin-dependent PI3K-Akt signaling. Insulin binds to the receptor and causes its phosphorylation, then IRS, PI3K and PKB are also phosphorylated. In a further step, PKB leads to an increased expression and translocation of GLUT4 to the cell membrane and increased glucose uptake [26,117,118]. The same study also proved that magnolol at a dose of 100 mg/kg reduced the level of TC and glucose in the blood plasma, caused the increase in mRNA expression for PPARγ, increased GLUT4 and adiponectin expression for targeted PPARγ genes and presumably increased glucose uptake by activation of the Akt-GLUT4 pathway [26]. There is also a molecular study performed with the use of luciferase assay indicating that magnolol, apart from being a ligand for PPARγ, can be also a ligand for RXRα [119].

It was proven that in the in vitro model magnolol promotes the differentiation of adipocytes in 3T3-L1 preadipocytes and C3H10T1/2 pluripotent stem cells. This is associated with increased expression of CCAAT/enhancer-binding protein delta (C/EBPδ), CCAAT/enhancer-binding protein alpha (C/EBPα) (which both are transcription factors) and PPARγ2 in the early phase of adipocyte differentiation in 3T3-L1 preadipocytes. Furthermore, it was found that magnolol-treated 3T3-L1 preadipocytes showed an increased mRNA synthesis of adipocyte Protein 2, adiponectin and lipoprotein lipase (LPL), necessary for the expression of the adipocyte phenotype. At a concentration of 10 μM, magnolol resulted in an increased insulin-dependent glucose uptake and increased mRNA expression of GLUT1 and GLUT4 in 3T3-L1 adipocytes [13].

Another research indicates that in vitro uptake of a fluorescent glucose analog 2-NBDG, which is used to monitor glucose uptake in living insulin-sensitive cells, in murine 3T3-F442A preadipocytes and human subcutaneous adipocytes is magnolol dose-dependent. *Magnolia dealbata* ethanolic extract was used in that study, and magnolol and honokiol were the two main active compounds found in it. It was emphasized that magnolol influences the stimulation of glucose uptake in insulin-sensitive and insulin-insensitive mice and human adipocytes through insulin-signaling pathway [10].

Administration of magnolol at a dose of 17 mg/kg to mice on a HFD reduced the size of adipocytes as adipogenesis was inhibited. There was a significant reduction of fatty acid synthase (FAS) activity, and differentiation of adipocytes in epididymal white adipose tissue. Also, the mRNA expression of the key genes responsible for the synthesis and uptake of fatty acids was lowered. In this study, magnolol showed a two-fold protective effect against the development of insulin resistance under the influence of HFD by downregulating the expression of pro-inflammatory genes in visceral white adipose tissue and increasing the level of IL-10 (a factor with anti-inflammatory properties) in the plasma. It is worth mentioning that magnolol treatment resulted also in a significant decrease in homeostatic model assessment of insulin resistance (HOMA-IR) in HFD-fed mice, although the fasting blood glucose and plasma insulin levels were not significantly altered. Moreover, in the intraperitoneal glucose tolerance test, the blood glucose level was significantly reduced by magnolol and the AUC was lower than AUC obtained from untreated animals [11].

The effect of magnolol on the browning process of adipocytes was also investigated [14], since brown adipose tissue, as opposed to white, can be potentially helpful in the process of counteracting obesity and reducing complications resulting from it [120]. The process of adipocytes browning can be triggered by supplementation with natural antioxidant compounds, and this is what magnolol is considered to be. To prove the effect of magnolol on browning of adipocytes, an in vitro study on the 3T3-L1 and HIB1 B lines of preadipocytes was carried out and it was shown that the treatment with this lignan had a positive effect on the improvement of parameters related to potential obesity. An improvement in the lipid metabolism by decreasing lipid content and decreasing the expression of lipogenic markers such as sterol regulatory element binding transcription factor 1 or FAS were observed. Moreover, the increase in lipid oxidation, increase in lipolysis, increase in expression of AMPK and the decrease in fatty acid synthesis was noted. Increased activities of superoxide dismutase (SOD) and catalase (CAT) suggesting an inhibition of oxidative stress were also detected. Importantly, an activation of browning of adipocytes exhibited by the activation of pathways mediated by AMPK, PPARγ and protein kinase A was also observed after magnolol pretreatment. In this experiment magnolol was tested at concentrations of 1, 5, 10, and 20 μM, and it was noticed that the higher the magnolol dose was, the greater effectiveness was observed, wherein, 20 µM dose showed no toxicity [14]. Verification for these reports can be found in another study [121].

Lipoprotein lipase (LPL) is one of the enzymes that are involved in the metabolism of lipids [122]. In order to analyze the effect of magnolol on the activity of LPL, both in vitro and in vivo tests were carried out. The in vitro stage was conducted on a cell culture of mouse 3T3-L1 preadipocytes and the in vivo part on a transgenic apolipoprotein A5 knock-in mice with an induced low plasma TG level with no effect on plasma cholesterol. These mice possessed a variant of the human c.553G> T allele, which is a variant responsible for the occurrence of hyperlipidemia and increased risk of coronary artery disease. The results obtained in the course of the study indicate a possible reduction in the postprandial plasma TG concentration in the apolipoprotein A5 c.553G>T variant carrier mice. Additionally, LPL activity was increased in both cell culture and mouse plasma [123].

Furthermore, in the HFD-induced diabetic rats, administration of magnolol resulted in normalization of the lipid profile. The level of TG, TC and LDL in the serum was significantly reduced [21].

### 3.7. Magnolol and Reproductive System Disturbances

Polycystic ovary syndrome (PCOS) is a feminine endocrine disease. It is associated with pathological changes such as hyperandrogenism, chronic ovulation failure or infertility, menstruation disorders, endometrial hyperplasia, and metabolic disorders, especially obesity and insulin resistance (IR) [124,125,126,127]. IR is characterized by the abnormal body response to this hormone. What is more, in IR, insulin binding with the tyrosine kinase receptor may also be impaired. This loss of insulin action can lead to alterations of intracellular insulin-dependent pathways. For instance, the pathway regulated by the activation of PI3K, which is associated with an enhanced peripheral glucose linking and NO production in endothelium can be disrupted. Additionally, disturbances in the pathway linked to the activation of MAPK, enabling the proliferation of smooth muscle, monocyte migration, and PAI-1 activation can also occur. To confirm IR, several parameters can be used. In IR, increased HOMA-IR, decreased amount of sexual hormone binding protein or altered PPAR-γ and leptin-mediated signal functions are observed [124]. Consequently, PCOS often leads to the development of type 2 diabetes and severe cardiovascular changes, accompanied by the metabolic syndrome [124,125,126,127]. Concerning individual case, there are several ways to manage PCOS. These encompass single or combined therapies with oral contraception, antiandrogens and/or insulin sensitizers. The latter include, among others, thiazolidinediones, drugs acting through the PPAR-γ [125,128,129,130,131,132].

We managed to find a study that indicates a potentially beneficial effect of magnolol in PCOS. The study was conducted on dehydroepiandrosterone (DHEA)-induced rat model of PCOS and insulin resistance. After injections with DHEA continued for 28 days, mature female Sprague Dawley rats showed typical symptoms of impaired reproductive system and insulin homeostasis. Increased number of cystically dilated follicles with thicker granulosa, elevated levels of luteinizing hormone (LH), fasting insulin, HOMA-IR and decreased ovarian protein expression of PPARγ, IRS1 and PKB were noted. Oral administration of magnolol at a dose of 500 mg/kg for 28 days, resulted in the decrease in the level of LH, number of cystically dilated follicles, as well as increased ovarian protein expression for PPARγ, IRS1 and PKB. This suggests that magnolol improved the histological, hormonal and metabolic aspects of DHEA-induced PCOS [46]. In another study, the same animal model of PCOS and insulin resistance was treated with magnolia extract. The obtained results revealed that this extract led to the decrease in the serum insulin and testosterone levels and the increase in the ovarian expression of IRS1, PKB and PPARγ [63]. Both these studies demonstrate that magnolol and magnolia extract are natural PPAR-γ agonists [46,63].

As far as male reproductive system disorders in diabetes are concerned, to the best of our knowledge, there is no published evidence in this matter.

### 3.8. Intestinal Bacteria in Obesity and Diabetes

An important issue that is just being raised among the specialists in the field of diabetes is change of the intestinal microflora in the course of this disease. It must be highlighted that a properly developed intestinal microflora is responsible for maintaining immunity in the mucous membranes of the body, the development of lymphoid tissues associated with the intestine, and the promotion of adaptive immune responses to pathogens. Studies conducted on animal models with diabetes and with people suffering from type 1 or type 2 diabetes confirmed the relationship between the hosts’ microbiota and their health or disease [133]. The most frequently mentioned genera of bacteria associated with type 2 diabetes include *Bifidobacterium*, *Bacteroides*, *Faecalibacterium*, *Akkermansia* and *Roseburia* as bacteria that inhibit its progress, and *Ruminococcus*, *Fusobacterium* and *Blautia* as microorganisms promoting its occurrence [134].

So far, no studies have been conducted regarding the magnolol effect on the condition and diversity of the intestinal microbiota in the course of diabetes. An interesting study, however, was carried out on mice with HFD-induced obesity, which were administered honokiol at doses of 200–800 mg/kg. The aim of that study was to find whether honokiol influences the regulation of the composition of the intestinal microbiota and if it improves the obesity-related parameters. The results obtained seem promising—honokiol reduced the body weight, adipose tissue mass and the size of the adipocytes of examined mice. Further, it reduced insulin resistance and the level of pro-inflammatory cytokines in their serum. In the case of the intestinal microflora, honokiol had a beneficial effect on obtaining microbiota homeostasis increasing the amount of *Akkermansia* bacteria and reducing the amount of *Oscillospira* bacteria [135].

As far as magnolol is concerned, it seems important to mention the study carried out on mice given an anticancer drug, oxaliplatin. The administration of magnolol at doses of 75 and 300 mg/kg resulted in the inhibition of weight loss, alleviation of diarrhea symptoms and improvement of the histopathological parameters in the colon. The changes caused by oxidative stress were inhibited; there was an increase in the activity of SOD, glutathione peroxidase (GPx) and the increase in the level of glutathione, as well as the decrease in the level of NF-κB and pro-inflammatory cytokines. Additionally, magnolol contributed to the restoration of the normal intestinal microflora, disturbed by the administration of oxaliplatin [136].

Among other publications describing the interplay between lignans and gut microbiota, two more works are worth mentioning. The first indicated that type 2 diabetic women have lower levels of two dietary lignan metabolites transformed by gut microbiota, enterolactone and enterodiol, in their urine than healthy women. The authors of this study also concluded that higher level of enterolactone is associated with a lower risk of diabetes incidence [137]. The second mentioned study describes modulation of gut microflora and reduction of intestinal inflammation in humans after using dietary lignans [138].

Taking into account the current scientific reports, it can be presumed that magnolol may have a regulatory potential on the intestinal microflora also in the course of diabetes. Therefore, it is necessary to conduct research that will confirm or reject such a hypothesis.

### 3.9. Magnolol and Oxidative Stress and Inflammation in Diabetes

Magnolol acts as a free radical scavenger which was proven in numerous in vitro and in vivo studies [15,16,18]. As it was mentioned before, in diabetes oxidative stress occurs and is one of the main causes of diabetic complications [1,2,3,4]. In the study conducted on the diabetic rats it was shown that magnolol improved hepatic oxidative stress markers including reduction of hepatic MDA levels and increasing the activities of antioxidative enzymes such as SOD, CAT and GPx [21].

High blood glucose inevitably leads to deterioration in the functionality of the pancreas. The function of islet β cells is impaired, they degranulate and there is a decrease in insulin immunoreactivity as a result of oxidative stress [27]. The endogenous protective mechanisms of the organism against oxidative stress enclose the increased activity of antioxidant enzymes including, among others, GPx. Considering the fact that β cells of pancreatic islets have a lower capacity to break down hydrogen peroxide (one of the representatives of ROS) and show lower activity of endogenous antioxidant enzymes, in particular GPx, they are more prone to complications resulting from chronic oxidative stress [34,35,139]. In vitro treatment of rat pancreatic β cells with MGO led to the increase in GPx activity in these cells. This phenomenon can be explained by the fact that pancreatic cells coped with free radicals precisely by increasing expression for GPx. In this study, however, administration of magnolol (which is considered to be an antioxidant) resulted in a further increase in GPx expression. This is probably related to the role of the GPx enzyme itself in the body’s tolerance to oxidative stress. In relation to the above-mentioned study, magnolol significantly inhibited the production of ROS and reduced the oxidative damage caused by MGO thanks to the increased activity of GPx, in particular by reducing the levels of H_2_O_2_, O^2−^ and lipid hydroperoxide. As a conclusion, the authors of this study indicate that the administration of magnolol could have contributed to the reduction of the phenomenon of apoptosis of pancreatic islet β cells by increasing the expression of GPx [35].

Interleukin 1β (IL-1β) is a pro-inflammatory cytokine involved in the pathogenesis of both type 1 and type 2 diabetes. IL-1β is produced and secreted by the β cells of the pancreatic islets under conditions of high blood glucose and is believed to increase the level of ROS in the body [140,141]. The unfavorable effect of IL-1β on pancreatic β cells is manifested, among others, by inhibiting insulin transcription, reducing proinsulin conversion and inhibiting insulin gene transcription. In addition, IL-1β activates NF-κB, thus contribute to an even greater dysfunction of pancreatic islet β cells. In vitro pretreatment of rat pancreatic β cells exposed to MGO with magnolol resulted in a significant reduction in IL-1β levels and showed anti-inflammatory effects [35].

Equal parts of magnolia bark, pueraria, liquorice and spurge roots were used to prepare the ethanolic extract KIOM-4. The in vitro effect of this mixture against STZ-induced oxidative stress in rat pancreatic β-cells (RINm5F) was investigated. It turned out that KIOM-4 caused a depletion in ROS production, upregulation of CAT, and manganese SOD activity, protein expression, as well as Nrf2 upregulation. Furthermore, KIOM-4 treatment occurred to prevent STZ-induced mitochondrial lipid peroxidation, protein carbonyl formation and DNA modification. It re-established the loss of mitochondrial membrane potential, impeded the translocation of cytochrome c from the mitochondria to the cytosol, raised the level of ATP, succinate dehydrogenase activity and insulin level. KIOM-4 also reduced the STZ-induced endoplasmic reticulum stress and apoptosis in the RINm5F cells. The KIOM-4 extract also protected the RINm5F cells from lipid peroxidation and DNA damage caused by STZ. Moreover, the behavior of KIOM-4 as a free radical scavenger in cell-free chemical system was investigated. KIOM-4 scavenged superoxide and hydroxyl radicals generated by xanthine/xanthine oxidase, as well as Fenton reaction. This extract also revealed a scavenging activity towards the 1,1-diphenyl-2-picrylhydrazyl radical and STZ-induced intracellular ROS [60,61,142].

Periodontal disease is a long-term inflammation state that leads to destruction of the underlying tissues that support the teeth. It was observed that patients suffering from periodontitis and diabetes simultaneously, show a worse prognosis than those who struggle only with the above-mentioned inflammation [45,143]. In the course of these diseases, increased expression of periodontal IL-6 and plasma IL-8 is observed [144,145]. Moreover, patients with advanced form of periodontitis experienced a downregulation of nuclear factor erythroid 2-related factor 2 (Nrf2) in oral neutrophils [146]. A reduced expression of heme oxygenase-1 (HO-1), one of its major target genes, is also often noticed [147,148]. It was indicated that AGEs play a significant role in tissue destruction and the disruption of remedial actions in periodontitis accompanied by diabetes. Inflammation in the course of periodontitis is caused not only by bacterial biofilm and increased production of ROS, but also by the state of hyperglycemia, which leads to the phenomenon of oxidative stress in the gingiva [149,150,151]. An in vitro study on the human gingival fibroblasts line to which AGEs were added at a concentration of 500 µg/mL revealed that there were adverse changes in these cells. An increased production of ROS, as well as IL-6 and IL-8, and the decrease in the ability to heal wounds with the accompanying depletion in the expression of the signal pathway for Nrf2/HO-1 were observed. Administration of magnolol, in concentrations of 2.5, 5, and 10 mM, respectively, resulted in a reversal of all unfavorable changes that occurred under the influence of AGEs. Overall, magnolol was able to improve wound healing capacity through downregulation of IL-6 and IL-8 via upregulation of Nrf-2/HO-1 pathway [45].

### 3.10. Summary of Beneficial Effects of Magnolol in the Course of Diabetes and Diabetic Complications and Comorbidities

**Figure 2:**
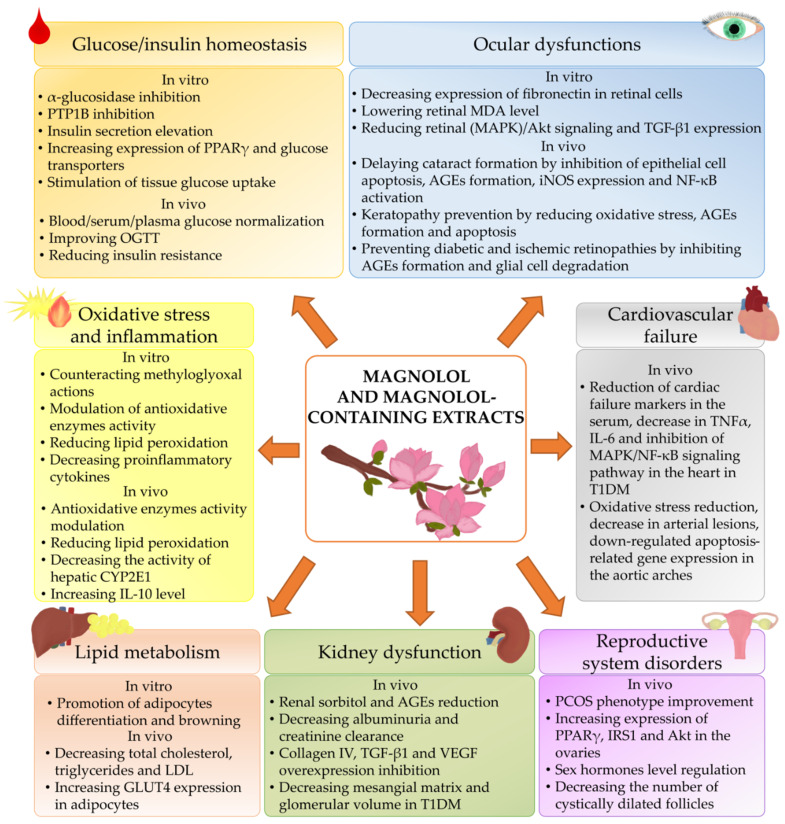
Effect of magnolol and magnolol-containing extracts on diabetes and diabetes-related condition. AGEs, advanced glycation end products; Akt, protein kinase B; CYP2E1, cytochrome P450 2E1; GLUT4, glucose transporter 4; IL-6, interleukin 6; IL-10, interleukin 10; iNOS, induced nitric oxide synthase; IRS1, insulin receptor substrate 1; LDL, low-density lipoprotein; MAPK, mitogen-activated protein kinase; MDA, malondialdehyde; NF-κB, nuclear factor kappa-light-chain-enhancer of activated B cells; OGTT, oral glucose tolerance test; PCOS, polycystic ovary syndrome; PPARγ, peroxisome proliferator-activated receptor gamma; PTP1B, protein tyrosine phosphatase-1B; T1DM, type 1 diabetes mellitus; TGF-β1, transforming growth factor β1; TNFα, tumor necrosis factor α; VEGF, vascular endothelial growth factor.

Beneficial effects of magnolol and magnolol-containing extracts on diabetes and diabetes-related condition are summarized in the Figure 2.

## 4. Conclusions

In this study, we tried to present the current state of knowledge regarding the effect of magnolol on the course of diabetes and its complications based on the animal studies. The scientific reports, that we compiled here, confirm that magnolol is a multifunctional agent which can be helpful in management of type 2 diabetes, its complications and comorbidities. Magnolol reveals antidiabetic properties, improves insulin tolerance and normalizes glucose homeostasis, prevents obesity, and protects the body against oxidative stress. It also shows beneficial activities in diabetic cataract, retinopathy and nephropathy. This lignan may be also a beneficial agent in the treatment of endocrine disorders connected with disturbed insulin homeostasis such as PCOS. Although we have not come across any publications regarding its beneficial effects on circulatory disorders or neuropathy strictly connected with the course of type 2 diabetes (i.e., diabetic cardiomyopathy, disorders of autonomic functions and micro- and macro- vascular changes leading to disorders of male sexual function, diabetic polyneuropathies or diabetic foot), we briefly presented studies on the effects of magnolol, its combination with honokiol, or just honokiol (as its optical isomer) on disorders such as Parkinson’s disease, Alzheimer’s disease, depression, memory problems, ischemic stroke, cardiac fibrosis and arterial hypertension. Furthermore, we indicated the links between disturbed intestinal microbiota and the occurrence of diabetes, as well as the beneficial effect of lignans on restoring the state of intestinal homeostasis. Summarizing, magnolol is a plant-derived, natural compound that shows promising effects in the prevention and treatment of type 2 diabetes and its complications. Therefore, after the manifestation of such diseases, magnolol may be a good supportive compound complementing conventional therapeutic methods in counteracting diabetic negative effects on the organism.

## Figures and Tables

**Table 1:** Antidiabetic activity and the effect of magnolol on the basic parameters of oxidative stress in in vitro studies.

Model Line	Effect	Magnolol Dosage	Reference
Parameters of Glucose and Insulin Homeostasis			
Human retinal pigment epithelial cells ARPE-19 + high glucose concentration	decrease in glucose level	0.5–5 μg/mL pretreatment	[44]
Rat pancreatic β-cells RINm5F cells + MGO	increase in insulin secretion	0.01–1.0 µM pretreatment	[35]
α-glucosidase inhibition activity assay	decrease in α-glucosidase activity	magnolol 1.5 μM dimeric neolignan derivatives 1.5 μM	[31]
Enzymatic assay for PTP1B	decrease in PTP1B activity	1–250 μM	[43]
3T3-L1 preadipocytes + primary polyclonal antibodies anti-PPARγ	increase in mRNA of PPARγ expression	5, 10, 20 μM preatreatment	[14]
3T3-L1 adipocytes	increase in GLUT 1, GLUT 4 and GLUT 4 protein expression, increase in mRNA of PPARγ expression and in glucose uptake	10 μM	[13]
Murine 3T3-F442A and human subcutaneous adipocytes	increase in glucose uptake	30 μM	[10]
Parameters Related to Oxidative Stress, Inflammation and Molecules Glycation			
3T3-L1 preadipocytes + carboxy-H2 DCFDA fluorescent stain	increase in SOD and CAT activity	5, 10, 20 μM pretreatment	[14]
Rat pancreatic β-cells RINm5F cells + MGO	increase in GPx activity, decrease in IL-1β level	0.01–1.0 µM pretreatment	[35]
Human retinal pigment epithelial cells ARPE-19 + high glucose concentration	decrease in MDA level	0.5–5 μg/mL	[44]
Human gingival fibroblasts + (AGE)-BSA	decrease in AGEs, IL-6 and IL-8 levels	2.5, 5, 10 mM	[45]

**Table 2:** Antidiabetic activity and the effect of magnolol on the basic parameters of oxidative stress and lipids in in vivo studies.

Animal Model	Effect	Magnolol Dosage	Reference
Parameters of Glucose and Insulin Homeostasis			
HFD + STZ rats	decrease in glucose level in the serum	25, 50, 100 mg/kg once a day for 10 weeks	[21]
Type 2 diabetic Goto-Kakizaki rats	decrease in glucose level in the fasting blood	100 mg/kg once a day for 13 weeks	[27]
HFD C57BL/6J mice	decrease in glucose level in the fasting blood	0.02% (17 mg/kg) with HFD for 16 weeks	[11]
HFD + STZ rats	increase in glucose tolerance in the serum—OGTT	25, 50, 100 mg/kg once a day for 10 weeks	[21]
HFD obese mice	increase in glucose tolerance in the plasma—OGTT	100 mg/kg magnolol once a day for 8 weeks	[26]
HFD C57BL/6J mice	increase in glucose tolerance in the fasting blood—intraperitoneal glucose tolerance test and decrease in HOMA-IR in the fasting blood	0.02% (17 mg/kg) with HFD for 16 weeks	[11]
Type 2 diabetic Goto-Kakizaki rats	decrease in sorbitol level in the kidney medulla	100 mg/kg once a day for 13 weeks	[27]
Type 2 diabetic Goto-Kakizaki rats	decrease in insulin level in the fasting plasma	100 mg/kg once a day for 13 weeks	[27]
HFD C57BL/6J mice	decrease in insulin level in the fasting plasma	0.02% (17 mg/kg) with HFD for 16 weeks	[11]
Post-pubertal female Sprague Dawley rats + DHEA	increase in mRNA of PPARγ expression in the ovaries	500 mg/kg for 28 days	[46]
HFD obese mice	increase in mRNA of PPARγ expression and GLUT 4 expression in the white adipose tissue	100 mg/kg magnolol once a day for 8 weeks	[26]
Parameters of Lipid Metabolism Homeostasis			
HFD + STZ rats	decrease in TG, TC and LDL levels in the serum	25, 50, 100 mg/kg once a day for 10 weeks	[21]
HFD obese mice	decrease in TC level in the plasma	100 mg/kg magnolol once a day for 8 weeks	[26]
Parameters Related to Oxidative Stress, Inflammation and Molecules Glycation			
HFD + STZ rats	increase in SOD, CAT and GPx activities, decrease in MDA level and CYP2E1 activity in the liver	25, 50, 100 mg/kg once a day for 10 weeks	[21]
Type 2 diabetic Goto-Kakizaki rats	decrease in AGEs level in kidney glomeruli	100 mg/kg once a day for 13 weeks	[27]
HFD C57BL/6J mice	increase in IL-10 level in the plasma	0.02% (17 mg/kg) with HFD for 16 weeks	[11]

**Table 3:** (**a**) Antidiabetic activity and the effect of *Magnolia* extracts on the basic parameters of oxidative stress in in vitro studies. (**b**) Antidiabetic activity and the effect of *Magnolia* extracts on the basic parameters of oxidative stress and lipids in in vivo studies.

(**a**)			
**Model Line**	**Effect**	***Magnolia* Extracts Dosage**	**Reference**
Parameters of Glucose and Insulin Homeostasis			
Murine 3T3-F442A and human subcutaneous adipocytes	increase in glucose uptake	*Magnolia dealbata* extract 50 μg/mL	[10]
Parameters Related to Oxidative Stress, Inflammation and Molecules Glycation			
RINm5F rat pancreatic β-cells + STZ	increase in SOD activity	50 μg mL^−1^ KIOM-4 pretreatment	[60]
Rat pancreatic β-cells RINm5F cells + Triton X-100	increase in CAT activity, decrease in thiobarbituric acid reactive substances level	KIOM-4 pretreatment	[61]
In vitro	decrease in AGEs level	25–100 µg/mL unprocessed or processed magnolia bark extract	[57]
(**b**)			
**Animal Model**	**Effect**	***Magnolia* Extracts Dosage**	**Reference**
Parameters of Glucose and Insulin Homeostasis			
Zucker diabetic fatty rats	decrease in glucose level in the serum	50 mg/kg KIOM-79 once a day for 13 weeks	[54]
Type 2 diabetic Goto-Kakizaki rats	decrease in glucose level in the fasting blood-plasma	500 mg/kg KIOM-79 once a day for 13 weeks	[55]
Zucker diabetic fatty rats	decrease in glucose level in the fasting blood	50 mg/kg KIOM-79 once a day for 13 weeks	[53,62]
HFD obese mice	increase in glucose tolerance in the plasma—OGTT	50 + 50 mg/kg honokiol + magnolol once a day for 8 weeks	[26]
Type 2 diabetic Goto-Kakizaki rats	decrease in insulin level in the fasting plasma	500 mg/kg KIOM-79 once a day for 13 weeks	[55]
HFD obese mice	increase in mRNA of PPARγ expression and GLUT 4 expression in the white adipose tissue	50 + 50 mg/kg honokiol + magnolol once a day for 8 weeks	[26]
Post-pubertal female Sprague Dawley rats + DHEA	increase in mRNA of PPARγ expression in the ovaries	*Magnolia officinalis* extract for 28 days	[63]
Parameters of Lipid Metabolism Homeostasis			
Zucker diabetic fatty rats	decrease in TG and LDL levels in the plasma	50 mg/kg KIOM-79 once a day for 13 weeks	[54]
HFD obese mice	decrease in TC level in the plasma	50 + 50 mg/kg honokiol + magnolol once a day for 8 weeks	[26]
Parameters Related to Oxidative Stress, Inflammation and Molecules Glycation			
Zucker diabetic fatty rats	decrease in MDA level in the serum	50 mg/kg KIOM-79 once a day for 13 weeks	[54]
Zucker diabetic fatty rats	decrease in AGEs level in the kidney glomeruli and tubulointerstitium	50 mg/kg KIOM-79 once a day for 13 weeks	[54]
Type 2 diabetic Goto-Kakizaki rats	decrease in AGEs level in the kidney glomeruli and tubules	500 mg/kg KIOM-79 once a day for 13 weeks	[55]
Zucker diabetic fatty rats	decrease in AGEs level in the lens sections	50 mg/kg KIOM-79 once a day for 13 weeks	[53]
Zucker diabetic fatty rats	decrease in AGEs level in the cornea	50 mg/kg KIOM-79 once a day for 13 weeks	[62]
C57BL/KSJ-db/db mice	decrease in AGEs level in the retina	150 mg/kg KIOM-79 once a day for 12 weeks	[64]

## Abbreviations

AGEs	advanced glycation end products
AGIs	α-glucosidase inhibitors
AMPK	adenosine monophosphate-activated protein kinase
AUC	area under the curve
CAT	catalase
C/EBP (α/δ)	CCAAT/enhancer-binding protein (alpha/delta)
CYP2E1	cytochrome P450 2E1
DHEA	dehydroepiandrosterone
FAS	fatty acid synthase
GLUT (1/4)	glucose transporter (1/4)
GPx	glutathione peroxidase
HFD	high-fat diet
HO-1	heme oxygenase-1
HOMA-IR	homeostatic model assessment of insulin resistance
iNOS	induced nitric oxide synthase
IL (1β/6/10)	interleukin (1β/6/10)
IR	insulin resistance
IRS (1/2)	insulin receptor substrate (1/2)
LDL	low-density lipoprotein
LPL	lipoprotein lipase
MAPK	mitogen-activated protein kinase
MDA	malondialdehyde
MGO	methylglyoxal
mRNA	messenger ribonucleic acid
NFκB	nuclear factor kappa-light-chain-enhancer of activated B cells
NO	nitric oxide
Nrf2	nuclear factor erythroid 2-related factor 2
OGTT	oral glucose tolerance test
PAI-1	plasminogen activator inhibitor-1
PCOS	polycystic ovary syndrome
PI3K	phosphatidylinositol 3-kinase
PKB (=Akt)	protein kinase B
PPAR-γ	peroxisome proliferator-activated receptor gamma
PTP1B	protein tyrosine phosphatase-1B
ROS	reactive oxygen species
RXR	retinoid X receptor
SOD	superoxide dismutase
STZ	streptozotocin
T1DM	type 1 diabetes mellitus
TC	total cholesterol
TG	triglycerides
TGF-β	transforming growth factor β
TNF-α	tumor necrosis factor α
VEGF	vascular endothelial growth factor
